# Hope and Fear: A Survey of Eco-Emotions and Climate Anxiety, Activism, and Well-Being Among Older Adolescents in Northern California

**DOI:** 10.3390/ijerph23070834

**Published:** 2026-06-25

**Authors:** Kelly L. L’Engle, Julianna Sahoo, Gwendolyn M. Hoff Anderson, Elise Brown, Lexi Nutkiewicz

**Affiliations:** 1 School of Nursing and Health Professions, University of San Francisco, San Francisco, CA 94117, USA; jdrew2@usfca.edu (J.S.); gmhandersonmph@gmail.com (G.M.H.A.); ebrown@uwsp.edu (E.B.); lexi.nutkiewicz@cuanschutz.edu (L.N.); 2 School of Health Sciences and Wellness, University of Wisconsin-Stevens Point, Stevens Point, WI 54481, USA; 3 Department of Environmental and Occupational Health, Colorado School of Public Health, University of Colorado at Anschutz, Aurora, CO 80045, USA

**Keywords:** eco-anxiety, positive emotions, mental health, climate change, well-being, youth, adolescents, adolescence, young people

## Abstract

**Highlights:**

**Public health relevance—How does this work relate to a public health issue?**

- Climate change is a significant public health threat, with both physical and mental health implications for adolescents. The health impacts of climate change are wide-ranging, including exposure to pollution, disease, trauma related to a severe weather event, and the mental health impacts of losing a home, community, or disruption to a routine during adolescence.
- Adolescence is a critical developmental window, with unique risks and strengths.

**Public health significance—Why is this work of significance to public health?**

- Adolescents are already at risk for mental health conditions. Climate change adds an additional mental health burden.
- Climate activism is important to combat climate change but can also be detrimental to mental health.

**Public health implications—What are the key implications or messages for practitioners, policy makers and/or researchers in public health?**

- More research is necessary on how to motivate activism without negatively impacting mental health.
- Climate activist messaging is more effective when the impact on mental health is considered. Bolstering positive emotions, such as determination and motivation, may be a powerful way to support activism while also protecting mental health in adolescents.

**Abstract:**

The purpose of this study is to examine positive and negative emotions about climate change reported by youth living in northern California and explore how these emotions are linked to climate anxiety, activism, and other measures of well-being. We surveyed ethnically diverse first- and second-year students (N = 521, mean age = 19) at a Jesuit, urban university in California in Fall 2022. Survey measures assessed climate-related emotions, eco-anxiety, and eco-impairment, along with activism, optimism, and compassion. Bivariate and multivariate models examined positive and negative eco-emotions, controlling for race, gender, and income. Overall, climate anxiety was linked to greater activism and confidence that actions matter. However, experiencing positive climate-related emotions had a stronger relationship to activism and optimism for the present and future, compared to negative emotions which were linked to higher eco-anxiety and greater compassion for others. Climate education and communication should consider inducing and reinforcing positive emotions to encourage youth activism, especially since negative emotions in response to climate change are linked to worse mental health. More research on a range of climate emotions is needed, and future interventions should test how to induce hope without minimizing the seriousness of climate change to support confidence and youth action.

## 1. Introduction

### 1.1. Youth Susceptibility to the Climate Crisis

Young people are uniquely susceptible to climate change. UNICEF calls children and youth “climate changed” [1]; the climate crisis is not just changing the planet but changing young people as well. Climate change and related severe weather events result in exposure to harmful air pollution, extreme and prolonged heat waves, increased risk of diseases and pandemics, and physical injuries from natural disasters. In a 2023 survey, 93% of 16–25-year-olds in the United States (U.S.) reported exposure to at least one severe weather event in the past year [2]. Due to the rapid physical, mental, and emotional growth of adolescents, they are particularly vulnerable to negative consequences from climate change during this critical developmental window [3,4,5]. Climate change-related weather events contribute to a multitude of health conditions, including disease related to a lack of clean water after flooding, heat-related illnesses and chronic conditions exacerbated by heat, and respiratory illness worsened by wildfires and longer pollen seasons [6,7,8]. Displacement and loss of or harm to homes, schools, and neighborhoods resulting from climate events can lead to trauma, which increases lifelong risk for developing a mental health condition [4,9]. In addition, climate change threatens protective factors for adolescent well-being, such as safe play, education, stable routines, and supportive community environments, thereby compounding risks to healthy development [9]. While climate change strongly impacts young people, they lack power and control over their environment and question the impact of their actions [10,11,12]. Climate change has been referred to as a “threat multiplier” for lower-income and minority youth in particular because they face a greater risk of experiencing the direct effects of climate change, exposure to environmental hazards, and exacerbations of existing health disparities [13].

### 1.2. Anxiety About Climate Change

Beyond physical, social, and environmental consequences, climate change is increasingly recognized as a threat to young people’s mental health and well-being. These include heightened levels of stress, fear, and worry regarding the deterioration of the environment [14], the perception of diminished future opportunities due to climate change [15], and functional impairments in sleep, eating, and socializing as a consequence of climate-related thoughts [15,16]. Among youth in California, feeling nervous, depressed, or stressed about climate change has been associated with higher rates of psychological distress and suicidal ideation [17].

Climate anxiety among adolescents is high and may be increasing. A 2021 global study of youth from 10 countries found that 59% were very or extremely worried about climate change, and 45% believed that their feelings about climate change negatively impacted their daily life activities. Three-quarters (75%) said that the future is frightening, more than half (55%) believe they won’t have the same opportunities as their parents, and 39% reported they are hesitant to have children due to climate change [15]. A 2023 survey of young adults in the U.S. found that 85% were worried about climate change and its impact on people and the planet. In the same survey, 38% said their feelings about climate change are negatively impacting their daily life activities, such as focusing on school, having fun, sleeping, or enjoying their friends, and 43% said that climate change is negatively impacting their mental health [2]. In a 2021 survey of California adolescents, 38% reported feeling nervous, depressed, or emotionally distressed due to climate change [18].

### 1.3. Emotions and Climate Activism

Although emotional responses to climate change are considered an essential element of climate anxiety, few scholars have conducted an in-depth examination of the range of emotions that young people feel about the climate crisis. People also may have positive feelings directed toward tackling the climate crisis, such as hope for a better future, feeling empowered while advocating for change, or a sense of community due to collective action [10]. Negative feelings are common and consistently associated with climate activism [14,19,20]. However, investigating and normalizing the range of emotions about climate change—both negative and positive—may help to increase resilience and drive among young people tackling climate change [12,21] and inform motivational strategies for increasing pro-environmental behaviors [22].

Although negative feelings about the climate crisis can motivate activism, they negatively impact well-being [14]. Instead of exacerbating negative feelings among youth, fostering positive emotions such as determination and empowerment around the climate crisis may motivate action without harming and possibly improving mental health. Student activists report a range of emotions that include disadvantages to taking action, such as feeling guilty and exhausted, but they also recognize the benefits of activism, such as gaining a sense of community, purpose, and identity [23]. Research with adolescents has found that perceiving high levels of social support and social connectedness may contribute to developing resilience [24], and activism contributes to positive youth development by building self-regulation skills for emotions and behaviors [25]. Finding ways to support hope and optimism alongside the community, identity, and skill-building aspects of climate activism may help to keep youth engaged while also protecting their mental health and contributing to positive youth development.

In response to government inaction and concern for their futures, young people have emerged as leading voices on climate change and mobilized the largest climate movement in history [25]. Investigating the broader emotional landscape of climate change and how both positive and negative feelings relate to activism and well-being could inform more effective public health communication and mental health support strategies for young people. This research will help to discern communication and education frameworks that may alleviate climate anxiety while encouraging young people to work towards securing a better future.

### 1.4. Study Purpose

California has a youth population that is multi-cultural and increasingly exposed to weather-related events such as severe storms, extreme heat, and wildfires [17,26]. The U.S. population is growing in racial and ethnic diversity, so how diverse California youth experience climate change and climate distress may capture these emotions and experiences among youth living in multi-cultural communities in other states and countries. With the increasing public health risks posed by climate change, empowering those who are most affected is crucial. The purpose of this study is to examine the range of emotions about climate change reported by youth living in northern California, including both positive and negative feelings about the climate crisis, and explore how these emotions are linked to climate anxiety, activism, and other measures of well-being.

## 2. Methods

### 2.1. Participants and Procedures

We conducted a cross-sectional online survey, distributed to all first- and second-year undergraduate students at an urban, Jesuit university in northern California in the Fall of 2022. The survey was created using a web-based survey tool, Qualtrics (2022, qualtrics.com), to evaluate students’ feelings and attitudes toward climate change, activism on the environment, exposure to extreme weather events, and well-being. All eligible students were sent emails that included a hyperlink to the informed consent form and online survey for the study. Two reminder emails were sent to non-responders at one and two weeks following the initial email. Students who completed the survey were offered the opportunity to enter a lottery to win a $50 gift card. Informed consent was obtained from study participants before beginning the survey. All responses to the survey were anonymized, and participant names and emails were not linked to their responses.

A total of 521 (17% of invited students) completed the survey. Approximately four out of five respondents were 17–19 years old (80%; mean age = 18.9, age range 17–24), female (78%; 16% male; 5% nonbinary), and students of color (78% non-white), see (Supplemental File S1). Almost one-third (31%) were finding it difficult to live on their present income, and four out of ten (39%) were first-generation college students. Survey respondents were representative of all first- and second-year students at the university in terms of non-white race/ethnicity and first-generation college students, although females were overrepresented. The large majority of students (83%) reported extreme weather events in their community during the last year, which included exposure to extreme heat (68%), poor air quality (45%), wildfires (42%), and other weather events.

### 2.2. Measures

#### 2.2.1. Climate Anxiety

We assessed three elements of climate anxiety that refer to emotional, psychological, and functional impacts of climate change and are mentioned in the literature: eco-emotions, eco-anxiety, and eco-impairment [15]. Climate change emotions were introduced with the question, “When thinking about climate change, how strongly do you feel the following emotions?” A list of 15 emotions was provided, and each one was scored from 1 (very) to 4 (not at all) [15,19,21]. Exploratory factor analysis identified two main groups of emotions. Items in each group loaded onto a single factor, and individual items in each group had reliable factor loadings of 0.50 or above [27], providing strong statistical evidence for which items belong together and significantly contribute to the underlying factor. The six positive climate change emotions were hopeful, faithful, empowered, motivated, supported, and determined (⍺ = 0.80). The six negative climate change emotions were angry, guilty, disgusted, frustrated, afraid, and nervous (⍺ = 0.85). Two emotions—doubtful and indifferent—were loaded onto a third factor but were poorly correlated, and feeling interested loaded moderately on all factors; these three emotions were not included in the eco-emotion measures used in this study. Additional details on the exploratory factor analysis are provided in (Supplemental File S2).

An 8-item scale was utilized to measure eco-anxiety, expressed as believing that climate change negatively impacts future opportunities (⍺ = 0.82). The introduction question asked, “Does climate change make you think of any of the following?” and statements included “I am hesitant to have children,” “People have failed to take care of the planet,” and “The things I value most will be destroyed.” [15]. Response options were “yes,” “no,” or “prefer not to say” and were grouped into “yes” versus “no/prefer not to say” for presentation and analysis. Eco-impairment was measured as impact on daily functioning using a 3-item scale (⍺ = 0.84). Respondents were asked, “Over the last 2 weeks, how often has thinking about climate-related events and other global environmental conditions affected the following activities?” [15,16]. Activities included sleeping, enjoying time with family and/or friends, and working and/or studying, and were scored from 1 (not at all) to 4 (nearly every day).

#### 2.2.2. Climate Action

To assess positive beliefs about taking action on climate change, an 8-item scale (⍺ = 0.80) was used that included statements such as “My actions have an influence on climate change” and “My actions to reduce climate change will encourage others [28,29].” Response options were 1 (strongly disagree) to 4 (strongly agree). Climate activism was measured using a 12-item scale with the introduction question, “In the past 12 months, how often did you engage in the following activities?” (⍺ = 0.86). Activities included “advocacy related to the environment (e.g., joined a protest or demonstration, contacted politicians),” “read a book and/or watched something about the environment,” and “made a choice to not buy a certain product,” and were scored from 1 (never) to 4 (often).

#### 2.2.3. Optimism and Compassion

The 7-item Children’s Hope Scale (⍺ = 0.84) measured positivity and optimism and included items such as “I think I’m doing pretty well” and “When I have a problem, I can come up with ways to solve it.” [30]. Items were scored from 1 (all of the time) to 6 (none of the time). The 4-item compassion subscale (⍺ = 0.79), from the Dispositional Positive Emotion Scales questionnaire, included items such as “I often notice people who need help” and “It’s important to take care of people who are vulnerable [31].” Response options ranged from 1 (strongly disagree) to 4 (strongly agree).

### 2.3. Analysis

We created multi-item scales for all key measures by summing and averaging items to create the scale score. Higher scale scores indicate greater levels of the construct (e.g., more positive eco-emotions, higher eco-anxiety, frequent climate activism). All scales performed well in reliability testing, with Cronbach’s alpha measures of internal consistency ranging from 0.80 to 0.86, as seen in Table 1.

We used a two-stage analytical approach, moving from simpler descriptive comparisons to more complex statistical models. First, variations in positive and negative climate emotions by demographic characteristics were examined in bivariate analyses. Relationships between indicators of climate anxiety (eco-emotions, eco-anxiety, eco-impairment) and activism and well-being were then assessed with Pearson correlation coefficients (*r*), which measure the strength of the association between two variables.

Second, General Linear Models (GLM) were used to examine how eco-emotions were related to key variables while accounting for demographic characteristics of respondents. In these models, climate emotions were entered as covariates, and eco-anxiety, eco-impairment, activism, and well-being were treated as dependent variables (i.e., the outcomes of interest). A separate GLM model was conducted for each outcome, controlling for race, gender, and income. Positive and negative eco-emotion scales were entered simultaneously into each model, which allowed us to assess the overall contribution of climate emotions and to directly compare how positive versus negative feelings related to each outcome. Model assumptions were assessed using residual plots and measures of model fit; multicollinearity was assessed using Variance Inflation Factor (VIF) statistics, with all values close to 1, indicating that multicollinearity was not a problem in these models. Effect sizes (partial η^2^) were used to evaluate the strength of observed relationships, beyond just statistical significance. As this was a convenience sample, no a priori power calculation was conducted; however, the achieved sample size of 521 provides sufficient power to detect small-to-medium effects in the analyses we conducted.

Data analyses were conducted using SPSS v29 (IBM, Armonk, NY, USA), and listwise deletion was used to handle missing data.

The full measurement instrument is provided in (Supplemental File S3).

## 3. Results

### 3.1. Experiences of Climate Anxiety

**Figure 1:**
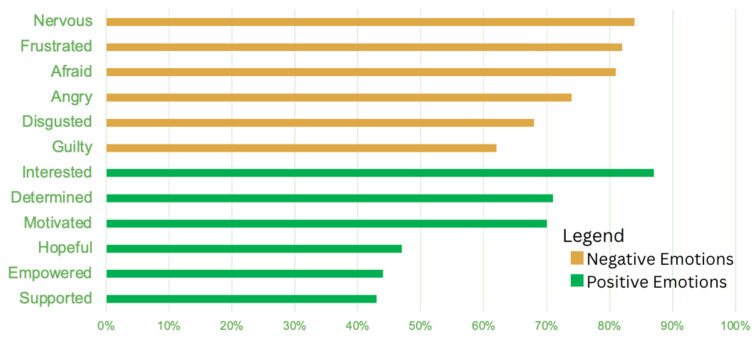
Eco-emotions: Chart shows the proportion of respondents who reported feeling the emotion “very” strongly or “moderately” strong when thinking about climate change.

Nervousness (84%) and frustration (82%) were the most common negative emotions felt when thinking about climate change, while determination (71%) and motivation (70%) were the most common positive emotions reported by respondents (Figure 1). Paired *t*-test results revealed that negative emotions about climate change (*M* = 3.15, *SD* = 0.62) were more frequently reported than positive emotions (*M* = 2.62, *SD* = 0.55), *t*(479) = 15.55, *p* < 0.001). In ANOVA results, female and non-binary students felt significantly more negative emotions about climate change than male students (*F*[2, 482] = 15.98, *p* < 0.001). Minority students felt significantly fewer negative emotions compared to peers (*F*[4, 481] = 8.77, *p* < 0.001). There were no statistically significant differences in negative emotions by age group or income or in positive emotions across sociodemographic groups (see Supplemental File S1). There was a small degree of covariation between positive and negative emotion scales, *r* = 0.17, *p* < 0.001. Overall, students reported a mix of negative and positive emotions about climate change. Although negative emotions were more common, many students also reported feeling determined and motivated.

Means in Table 1 show that eco-anxiety was high: on average, respondents endorsed five out of eight statements, indicating they believe that climate change is harming their future opportunities. Statements including “I’m hesitant to have children” (56%), “The things I value most will be destroyed” (56%), and “Humanity is doomed” (61%) were endorsed by more than half of the students. Conversely, eco-impairment was low in the sample (*M* = 1.46, *SD* = 0.61, range 1–4). Approximately four out of ten (42%) reported no functional impact from thinking about climate change, and fewer than 10% of students said that any of their daily activities were impacted on at least half of the days in the last two weeks. These findings indicate that while many students were concerned about the future impacts of climate change, relatively few reported that climate-related worries regularly interfered with their daily functioning.

### 3.2. Describing Activism, Optimism, and Compassion

Respondents reported mixed levels of climate activism (*M* = 2.21, *SD* = 0.58, range 1–4), with about half engaged in climate actions sometimes or often, but the other half rarely or never. The highest ranking climate actions were making a choice not to buy a certain product (72%), talking to other people about environmental issues (63%), and keeping up to date about climate news (63%); paired *t*-test results showed that personal actions like these were significantly more common than collective actions such as joining a climate protest (25%) or environmental organization outside of the college setting (18%), *t*(433) = 35.67, *p* < 0.001. On the other hand, students overall believed that climate activism makes a positive impact and contributes to improved mental health and well-being (*M* = 3.05, *SD* = 0.46, range 1–4). For example, 80% agreed that “My actions have an influence on climate change,” and 86% agreed that “Collective action will help combat climate change or environmental damage.” Most (82%) believed that climate change awareness has “given me more appreciation and a deeper connection to people and the planet,” 68% agreed that “Connecting with like-minded people around climate change has helped me feel empowered,” and 57% reported that “Addressing climate change or environmental damage helps me cope with my distress.” In summary, students were more likely to engage in individual lifestyle choices than formal collective activism, and the majority believed that both personal and collective action can make a real difference. Many students also viewed climate engagement as a source of empowerment and connection rather than solely a source of stress.

Respondents ranked in the middle on the optimism scale (*M* = 3.53, *SD* = 0.61; range 1–6): 65% believed a lot or all of the time that “The things I have done in the past will help me in the future,” but only 42% reported “I am doing pretty well” a lot or all of the time. However, compassion was high among students (*M* = 3.32, *SD* = 0.49; range 1–4), with about nine out of ten students saying they are a compassionate person who notices and helps vulnerable people. These results show that students generally viewed themselves as caring and compassionate while maintaining a more moderate level of optimism about their present and future circumstances.

### 3.3. Associations Between Eco-Emotions, Climate Anxiety, and Activism

Pearson correlation coefficients displayed in Table 1 indicate that positive eco-emotions were weakly related to eco-impairment (*r* = 0.11, *p* < 0.05) but unrelated to eco-anxiety. On the other hand, negative eco-emotions had a non-significant association with eco-impairment but a strong bivariate correlation with eco-anxiety (*r* = 0.57, *p* < 0.001). In the multivariable model controlling for gender, race, and income in Table 2, there remained a non-significant association between positive eco-emotions and eco-anxiety, while the association with negative eco-emotions remained substantial (partial η^2^ = 0.286, *p* < 0.001) once both types of emotions were considered. Positive eco-emotions remained weakly associated with eco-impairment in the multivariable model (partial η^2^ = 0.012, *p* = 0.019) while negative eco-emotions continued to show no association with eco-impairment. These findings suggest that students who experienced stronger negative emotions about climate change also reported substantially higher levels of climate anxiety, although they did not report greater disruptions in daily functioning. In contrast, positive eco-emotions were not associated with climate anxiety and showed only a weak relationship with eco-impairment.

Climate activism and believing that climate actions matter increased in tandem with both positive and negative feelings about climate change in bivariate analyses displayed in Table 1. In multivariable models in Table 3, positive eco-emotions showed stronger associations with climate activism (partial η^2^ = 0.095, *p* < 0.001) and confidence (partial η^2^ = 0.178, *p* < 0.001) compared to negative emotions (activism partial η^2^ = 0.061, *p* < 0.001, confidence partial η^2^ = 0.128, *p* < 0.001). The two measures of climate activism also showed small to moderate positive correlations with eco-anxiety and eco-impairment, as seen in Table 1. Overall, these results indicate that respondents who have stronger emotions about climate change and report greater climate anxiety are more environmentally engaged and confident that their actions will help to reduce environmental damage. Students who felt more strongly about climate change—whether positively or negatively—were more likely to take action. Notably, positive emotions were a somewhat stronger driver of both activism and confidence than negative emotions. This suggests that fostering hope and inspiration, not just urgency or fear, may be important for encouraging climate engagement.

### 3.4. Associations Between Optimism and Compassion with Key Measures

The pattern of results observed from bivariate and multivariate analyses indicates that optimism about the present and future is linked to positive emotions about climate change and believing that actions matter, while compassion is greatest among students who express negative emotions, high eco-anxiety, and frequent climate action. Students who were more optimistic felt stronger positive eco-emotions (*r* = 0.26, *p* < 0.001), but those with higher eco-anxiety (*r* = −0.14, *p* < 0.001) and eco-impairment (*r* = −0.13, *p* < 0.01) expressed significantly lower optimism as seen in Table 1. On the other hand, students who were more compassionate reported stronger negative emotions about climate change (*r* = 0.16, *p* < 0.001) and greater eco-anxiety (*r* = 0.13, *p* < 0.001). These findings held up in multivariable models displayed in Table 4: higher positive eco-emotions related to greater optimism (partial η^2^ = 0.063, *p* < 0.001), while stronger negative eco-emotions related to greater compassion (partial η^2^ = 0.018, *p* = 0.003). Compassion was also higher among respondents with higher levels of activism (r = 0.17, *p* < 0.001) and confidence that their actions will make a difference (*r* = 0.20, *r* < 0.001). In summary, students who felt hopeful and positive about climate change tended to be more optimistic generally, while students who felt angry or fearful tended to score higher on compassion.

### 3.5. Demographic Results in Multivariable Models

Results from the multivariable GLM models revealed some differences by demographic categories. Students who were having a difficult time living on their present income reported higher eco-anxiety and eco-impairment, but lower optimism. Males were lower on eco-anxiety, activism, and compassion and higher on optimism, compared to females and transgender/non-binary respondents; transgender/non-binary students scored most poorly on these four measures. These demographic patterns highlight that climate anxiety is not experienced equally. Students facing financial strain and transgender/non-binary students may be particularly vulnerable to climate-related distress and may benefit from additional support and resources.

## 4. Discussion

This is one of the first investigations to explore both positive and negative emotions about climate change reported by young people. We found that the range of eco-emotions mattered for understanding eco-anxiety and eco-impairment, levels of climate activism and confidence, and relationships with optimism and compassion. Although there is an increasing emphasis on centering youth voices in research, existing research has prioritized negative feelings and attitudes about the changing climate. This emphasis has left positive emotions, attitudes, and agency in confronting climate change less explored and not well understood [32,33]. Our study results make a compelling argument that positive feelings like hope and empowerment should receive greater attention to help alleviate negative mental health harms and encourage young people to take action to address the climate crisis [12]. Although our results showed that both positive and negative emotions were associated with climate activism in a sample of young college students in northern California, positive emotions were more strongly associated with climate activism and believing that climate actions will make a difference. These results align with emerging research showing that positive emotions can increase confidence and behavior change and foster resilience [11,22] and more established research documenting a link between negative emotions and climate activism [14,20].

Climate change is a difficult, complex issue that will require international cooperation, long-term thinking, and substantial behavior change to make meaningful progress [22]. As present-day youth will be the existing generation most affected by climate change, a better understanding of emotions and behaviors in this demographic is essential. Given the burden of negative emotions and climate change anxiety on mental health [34], there are ethical considerations to using negative emotions and fear appeals for advancing environmental causes [11,35]. Negative emotions can be debilitating and make taking action harder [11,14,16]. The adolescents we surveyed, similar to samples of U.S. adults, are aware of and concerned about climate change [15,19,36]. Youth in our sample overall felt more negative emotions around climate change than positive emotions. Adding stress and worry through constant negative communications to raise climate change awareness adds to a lifelong burden of chronic climate change anxiety [5].

Although previous research demonstrates that climate anxiety and negative emotions at moderate levels can motivate pro-environmental behaviors, many of these studies are limited and do not examine this effect over long time periods [37,38,39]. Additionally, communications that attempt to invoke negative feelings like fear or sadness can increase pro-environmental behaviors, such as donating to help the environment, but the impact may be short-lived [38,40]. In a study asking for donations to an environmental organization after watching either an emotionally neutral video or a sadness-inducing video, participants donated more on average immediately after watching the sad video. However, after simply introducing a one-hour delay between the video and request for donation, there was no difference in donation amount between the groups, suggesting a short-lived impact of emotional appeals that evoke sadness related to climate change [38]. Negative emotions are unpleasant, and since climate change does not have an easy solution, they may have a detrimental impact on climate activism and lead to hopelessness, apathy, and message avoidance [41].

A serious drawback in stimulating negative emotions in climate change communications is the potential for causing avoidance or denial, coping strategies used when people are presented with an emotional appeal but not provided a feasible plan of action or boosted self-efficacy [38,41,42]. In a study of pro-environmental behavior intentions in children, messaging that invoked empathy for animals while attributing blame to external factors led to avoidance and powerlessness [41]. When presented with a serious problem but no clear solution, adolescents tend to employ emotion-focused coping, such as avoidance and denial, which are detrimental to long-term well-being [43]. Believing that taking action will successfully mitigate climate change may be essential for motivating pro-environmental behaviors but also for channeling climate anxiety into climate action among young people; in a study of Filipino youth, higher climate anxiety was linked to greater climate action, but only among those who felt their actions would make a difference [12].

Inspiring, empowering, and motivating adolescents through positive emotions and a sense of agency is a promising approach to encourage pro-environmental behaviors and activism while protecting mental well-being. Motivating behavior change through positive emotions, instead of inducing negative feelings such as guilt and sadness, may lead to more productive engagement with climate change, including activism and information gathering [22]. Positively shaping communications and coping strategies can help mitigate the negative mental health impacts of climate change [22,44]. Positive emotions and their associated behaviors can be cyclical and thus beneficial in affecting long-term change [22]. Engaging youth long-term while shielding them from burnout associated with negative emotions may be a more sustainable approach to protecting youths’ mental health and well-being while confronting climate change.

The strong association we observed in the data correlating positive eco-emotions with believing that climate actions matter suggests a bidirectional relationship: engaging in climate activism may induce feelings of hope and empowerment, and the self-efficacy necessary for taking action and believing one’s efforts will make a difference likely sustains activism. An important question is whether positive eco-emotions reflect genuine agency, self-efficacy, or other factors that are not yet well understood; contributing factors to positive eco-emotions are likely multiple and complex, and our cross-sectional design does not allow us to identify them with confidence. Understanding what cultivates positive eco-emotions among youth remains an important direction for future research and for designing effective climate interventions and advocacy campaigns. Climate change advocacy does not require completely changing one’s lifestyle; instead, advocacy can be implemented through small daily decisions or conversations with friends and family that incrementally increase confidence and lead to greater activism. Like in other research, personal environmental actions were more common than collective actions; although some research has found that collective actions are key to reducing climate anxiety [10], more than half of the young people in our sample stated that taking action helps them cope with feeling distress from climate change. Personal actions may provide building blocks for confidence, connections, and skills leading to more community-focused collective action. The benefits of activism, including building a sense of community, purpose, and empowerment, may protect against some of the stress and exhaustion experienced by young activists [23]. Climate activism does not always require a substantial time commitment or additional stress, and focusing on the positive emotions associated with advocacy may foster mental health and well-being and sustain engagement in climate activism.

Strong negative feelings and high eco-anxiety in our sample of diverse young people in northern California mirror other research with youth in California and globally. Young people appear to be universally experiencing mental health harms from climate change, which are contributing to global feelings of despair, worry, and hopelessness about the future [15,45]. Concerns about climate change are likely impacting major life decisions [10]; people are increasingly concerned about the carbon footprint of having children and the well-being of their children in a climate-changed world [45]. Our findings that more than half of our young respondents said they are hesitant to have children and that humanity is doomed because of climate change provide insights into research showing that fertility plans are changing because of environmental concerns [45,46].

Notably, eco-impairment was low in our sample and this has been seen in other research with young adults [10], suggesting that negative thoughts and feelings may only be impacting daily functioning for a minority of youth. Scholars generally describe climate-related emotions as normal and adaptive responses to a real crisis rather than pathological states [4], and the low eco-impairment we observe may reflect adolescents’ capacity to hold serious climate concern without it substantially disrupting daily functioning. However, more vulnerable youth in our study sample, such as lower income and transgender and non-binary students, reported the highest eco-anxiety and eco-impairment and the least optimism among respondents; it may be that the “threat multiplier” of climate change is causing more severe mental health harms and a more harmful impact on daily life for the most vulnerable youth [13]. Hickman [47] posits gradations of increasingly harmful mental health impacts on young people from climate change, and it may be that the most vulnerable youth are more severely impacted and will experience harm earlier than peers. Additionally, our data suggest that even if climate change is not (yet) impacting daily activities, it is likely impacting youths’ life decisions and future plans. Although our study utilized existing and validated items and scales that demonstrated sound psychometric properties, there is robust discussion of the various dimensions and assessments of climate anxiety, and it is clear that more research and conceptual clarity are needed in this emerging field, especially with geographically, demographically, and culturally diverse samples [32,48].

Limitations of this study include a 17% response rate, which likely biased the data toward those with a higher interest in climate change. However, with increasing climate-related extreme weather events in California, the U.S., and globally, these data provide a snapshot of how young people are feeling about climate change and their well-being. Furthermore, although respondents were diverse in gender, race/ethnicity, and feelings about their income, the overall sample was predominantly female and from a Jesuit, private, urban university in California and may not be generalizable to other youth populations. For example, it is possible that compassion was higher in the study sample than levels that would be observed among students attending non-religious, non-Jesuit universities. Additionally, research shows that women and those with higher education are most concerned and distressed about climate change [32] and most likely to be engaged in climate activism [33,49], so it is possible that climate anxiety and activism would be lower in more gender-balanced or non-university samples of young people. Furthermore, the data are cross-sectional and therefore causality cannot be determined; it is probable that most of the key variables we investigated have bidirectional relationships, but longitudinal data would be able to answer key questions such as how to build positive eco-emotions and reduce negative feelings when confronted by and confronting climate change.

## 5. Conclusions

The results from this study indicate that more research on positive emotions, including longitudinal research and qualitative inquiry, could help to understand the relationship between climate anxiety, climate activism, social connection, and well-being among young people. Positive emotions show potential to have dual benefits of promoting mental health and well-being, along with supporting climate activism. Although negative emotions are linked with greater activism, the benefits may not outweigh the mental health burden that often comes with a pessimistic outlook on the changing climate. To mitigate this potential mental health burden, adolescent-oriented environmental education and climate messaging should include hope and agency to positively motivate activism.

Our findings could inform training for educators on productively communicating environmental health and climate change risks to adolescents, such that educators consider how to foster hope for young people without minimizing the seriousness of climate change. This can be implemented across educational topics and courses and facilitate accessible opportunities for young people to lead and take part in collective actions to combat climate change. Opportunities include inviting youth to discuss current climate solutions and participate in environmental action projects. As the changing climate continues to impact young people, facilitating positive emotions could improve their mental health and well-being.

## Figures and Tables

**Table 1:** Intercorrelations, means, standard deviation, and scale properties for key measures (N = 521).

	1.	2.	3.	4.	5.	6.	7.	8.
1. Positive eco-emotions	⍺ = 0.80							
2. Negative eco-emotions	0.17 ***	⍺ = 0.85						
3. Eco-anxiety	−0.00	0.57 ***	⍺ = 0.82					
4. Eco-impairment	0.11 *	0.02	0.09	⍺ = 0.84				
5. Climate activism	0.31 ***	0.33 ***	0.36 ***	0.30 ***	⍺ = 0.86			
6. Climate actions matter	0.44 ***	0.40 ***	0.31 ***	0.13 **	0.34 ***	⍺ = 0.80		
7. Optimism	0.26 ***	−0.04	−0.14 **	−0.13 **	0.00	0.14 **	⍺ = 0.84	
8. Compassion	0.04	0.16 ***	0.13 **	0.12 **	0.17 ***	0.20 ***	0.06	⍺ = 0.79
*M*	2.62	3.15	0.63	1.46	2.21	3.05	3.53	3.32
*SD*	0.55	0.62	0.30	0.61	0.58	0.46	0.61	0.49
Scale items, range	6, 1–4	6, 1–4	8, 0–1	6, 1–4	12, 1–4	8, 1–4	7, 1–6	4, 1–4

Pearson correlation coefficients. *M* = mean, *SD* = standard deviation. Reliability analyses (Cronbach’s alpha) are reported in the diagonal. * *p* < 0.05, ** *p* < 0.01, *** *p* < 0.001.

**Table 2:** Eco-anxiety and eco-impairment regressed on emotions about climate change: *F*-test and Eta-squared.

	Eco-Anxiety			Eco-Impairment		
	*F*	*p*	Partial η^2^	*F*	*p*	Partial η^2^
Positive eco-emotions	2.93	0.088	0.007	5.52	0.019	0.012
Negative eco-emotions	178.78	<0.001	0.286	0.00	0.975	0.000
Gender (control)	3.12	0.045	0.014	1.00	0.368	0.004
Race (control)	1.20	0.309	0.011	0.09	0.984	0.001
Income (control)	10.22	<0.001	0.022	9.33	0.002	0.020
Model df	11,447			11,452		
Model R^2^ (adjusted)	0.36			0.024		

General linear model (GLM) results shown for *F*-statistic, *p*-value, and partial eta-squared (η^2^) for each covariate and factor. All variables were entered simultaneously into the model.

**Table 3:** Activism regressed on emotions about climate change: *F*-test and Eta-squared.

	Climate Activism			Climate Confidence		
	*F*	*p*	Partial η^2^	*F*	*p*	Partial η^2^
Positive eco-emotions	42.49	<0.001	0.095	95.37	<0.001	0.178
Negative eco-emotions	26.40	<0.001	0.061	64.80	<0.001	0.128
Gender (control)	6.76	<0.001	0.032	1.82	0.164	0.008
Race (control)	2.41	0.049	0.023	2.09	0.081	0.019
Income (control)	6.44	0.012	0.016	1.01	0.316	0.002
Model df	11,407			11,440		
Model R^2^ (adjusted)	0.19			0.30		

General linear model (GLM) results shown for *F*-statistic, *p*-value, and partial eta-squared (η^2^) for each covariate and factor. All variables were entered simultaneously into the model.

**Table 4:** Optimism and compassion regressed on emotions about climate change: *F*-test and Eta-squared.

	Optimism			Compassion		
	*F*	*p*	Partial η^2^	*F*	*p*	Partial η^2^
Positive eco-emotions	31.02	<0.001	0.063	0.48	0.487	0.001
Negative eco-emotions	0.35	0.556	0.001	8.67	0.003	0.018
Gender (control)	8.29	<0.001	0.035	4.08	0.017	0.017
Race (control)	0.44	0.782	0.004	1.77	0.134	0.015
Income (control)	23.91	<0.001	0.050	6.03	0.014	0.013
Model df	11,459			11,460		
Model R^2^ (adjusted)	0.15			0.04		

General linear model (GLM) results shown for *F*-statistic, *p*-value, and partial eta-squared (η^2^) for each covariate and factor. All variables were entered simultaneously into the model.

## Data Availability

The dataset is not publicly available due to IRB requirements, but may be available upon request to the corresponding author.

## Supplementary Materials

The following supporting information can be downloaded at: https://www.mdpi.com/article/10.3390/ijerph23070834/s1, File S1: Background characteristics and emotions when thinking about climate change; File S2: Exploratory Factor Analysis; File S3: Survey Questions.

## References

[1] United Nations Children’s Fund (2023). The Climate-Changed Child: A Children’s Climate Risk Index Supplement.

[2] Lewandowski R.E., Clayton S.D., Olbrich L., Sakshaug J.W., Wray B., Schwartz S.E.O., Augustinavicius J., Howe P.D., Parnes M., Wright S. (2024). Climate Emotions, Thoughts, and Plans among US Adolescents and Young Adults: A Cross-Sectional Descriptive Survey and Analysis by Political Party Identification and Self-Reported Exposure to Severe Weather Events. Lancet Planet. Health.

[3] Nutkiewicz L., Habib A., Duenas M., Ruiz L., Anderson G.H., L’Engle K. (2023). Encouraging Youth Activism to Combat Climate Change and Reduce Eco-Anxiety. Int. Public Health J..

[4] Clayton S. (2020). Climate Anxiety: Psychological Responses to Climate Change. J. Anxiety Disord..

[5] Mosca A., Luciani D., Chiappini S., Miuli A., Cianconi P., Pettorruso M., Janiri L., Martinotti G., PsyClimate Research Group (2025). Eco-Anxiety and Mental Health: Correlates of Climate Change Distress. Int. J. Environ. Res. Public Health.

[6] Batra M., Erbas B. (2025). Extreme Weather, Vulnerable Populations, and Mental Health: The Timely Role of AI Interventions. Int. J. Environ. Res. Public Health.

[7] Leap S.R., Soled D.R., Sampath V., Nadeau K.C. (2024). Effects of Extreme Weather on Health in Underserved Communities. Ann. Allergy Asthma Immunol..

[8] Henning A., Kache S. (2025). Impact of the Climate Crisis on Childhood Health. Pediatr. Rev..

[9] Proulx K., Daelmans B., Baltag V., Banati P. (2024). Climate Change Impacts on Child and Adolescent Health and Well-Being: A Narrative Review. J. Glob. Health.

[10] Schwartz S.E.O., Benoit L., Clayton S., Parnes M.F., Swenson L., Lowe S.R. (2022). Climate Change Anxiety and Mental Health: Environmental Activism as Buffer. Curr. Psychol..

[11] Benoit L., Lowe S.R., Thomas I., Amsalem D., Martin A. (2025). Climate Change Hopefulness, Anxiety, and Behavioral Intentions among Adolescents: Randomized Controlled Trial of a Brief “Selfie” Video Intervention. Child Adolesc. Psychiatry Ment. Health.

[12] Aruta J.J.B.R. (2025). Climate Anxiety Mediates Environmental Concern and Climate Action among Filipino Youth: Does Mitigation Response Efficacy Belief Matter?. Curr. Psychol..

[13] Chalupka S., Anderko L., Pennea E. (2020). Climate Change, Climate Justice, and Children’s Mental Health: A Generation at Risk?. Environ. Justice.

[14] Ogunbode C.A., Doran R., Hanss D., Ojala M., Salmela-Aro K., Van Den Broek K.L., Bhullar N., Aquino S.D., Marot T., Schermer J.A. (2022). Climate Anxiety, Wellbeing and pro-Environmental Action: Correlates of Negative Emotional Responses to Climate Change in 32 Countries. J. Environ. Psychol..

[15] Hickman C., Marks E., Pihkala P., Clayton S., Lewandowski R.E., Mayall E.E., Wray B., Mellor C., Van Susteren L. (2021). Climate Anxiety in Children and Young People and Their Beliefs about Government Responses to Climate Change: A Global Survey. Lancet Planet. Health.

[16] Hogg T.L., Stanley S.K., O’Brien L.V., Wilson M.S., Watsford C.R. (2021). The Hogg Eco-Anxiety Scale: Development and Validation of a Multidimensional Scale. Glob. Environ. Change.

[17] Hindmarch G.M., Meza J., Shimkhada R., Padilla-Frausto I., Eisenberg D. (2025). Climate Change Stress Among Adolescents In California: Associations with Psychological Distress, Suicide Ideation, and Social and Demographic Factors. J. Adolesc. Health.

[18] Le A.D., Kelly S.W. (2024). 124. Rural vs. Urban Differences in Climate Change Anxiety and the Role of CIVIC Engagement Among California Adolescents. J. Adolesc. Health.

[19] Leiserowitz A., Maibach E., Rosenthal S., Kotcher J., Carman J., Neyens L., Myers T., Goldberg M., Campbell E., Lacroix K. (2022). Climate Change in the American Mind, April 2022.

[20] Weber E.U. (2006). Experience-Based and Description-Based Perceptions of Long-Term Risk: Why Global Warming Does Not Scare Us (Yet). Clim. Change.

[21] Duggan J., Haddaway N.R., Badullovich N. (2021). Climate Emotions: It Is Ok to Feel the Way You Do. Lancet Planet. Health.

[22] Schneider C.R., Zaval L., Markowitz E.M. (2021). Positive Emotions and Climate Change. Curr. Opin. Behav. Sci..

[23] Conner J.O., Crawford E., Galioto M. (2023). The Mental Health Effects of Student Activism: Persisting Despite Psychological Costs. J. Adolesc. Res..

[24] Cicek İ. (2021). Effect of Hope on Resilience in Adolescents: Social Support and Social Connectedness as Mediators. J. Posit. Sch. Psychol..

[25] Sanson A., Bellemo M. (2021). Children and Youth in the Climate Crisis. BJPsych Bull..

[26] California Department of Public Health. https://www.cdph.ca.gov/Programs/CFH/DMCAH/surveillance.

[27] Stevens J. (1992). Applied Multivariate Statistics for the Social Sciences.

[28] Kellstedt P.M., Zahran S., Vedlitz A. (2008). Personal Efficacy, the Information Environment, and Attitudes Toward Global Warming and Climate Change in the United States. Risk Anal..

[29] Overbury K. (2022). Research Portfolio Submitted in Part Fulfilment of the Requirements for the Degree of Doctorate in Clinical Psychology. Ph.D. Thesis.

[30] Snyder C.R., Hoza B., Pelham W.E., Rapoff M., Ware L., Danovsky M., Highberger L., Ribinstein H., Stahl K.J. (1997). The Development and Validation of the Children’s Hope Scale. J. Pediatr. Psychol..

[31] Shiota M.N., Keltner D., John O.P. (2006). Positive Emotion Dispositions Differentially Associated with Big Five Personality and Attachment Style. J. Posit. Psychol..

[32] Kırımer-Aydınlı F., Juaréz-Castelán M., Hakim N., Gul P., Unal A.B., Aguayo-Estremera R., Pérez-Fortis A., Rojas-Russell M.E., Gallo V. (2025). Measuring Planetary Eco-Emotions: A Systematic Review of Currently Available Instruments and Their Psychometric Properties. Curr. Psychol..

[33] Neas S., Ward A., Bowman B. (2022). Young People’s Climate Activism: A Review of the Literature. Front. Polit. Sci..

[34] Latkin C., Dayton L., Scherkoske M., Countess K., Thrul J. (2022). What Predicts Climate Change Activism?: An Examination of How Depressive Symptoms, Climate Change Distress, and Social Norms Are Associated with Climate Change Activism. J. Clim. Change Health.

[35] Pavani J.-B., Nicolas L., Bonetto E. (2023). Eco-Anxiety Motivates pro-Environmental Behaviors: A Two-Wave Longitudinal Study. Motiv. Emot..

[36] Ballew M.T., Leiserowitz A., Roser-Renouf C., Rosenthal S.A., Kotcher J.E., Marlon J.R., Lyon E., Goldberg M.H., Maibach E.W. (2019). Climate Change in the American Mind: Data, Tools, and Trends. Environ. Sci. Policy Sustain. Dev..

[37] Contreras A., Blanchard M.A., Mouguiama-Daouda C., Heeren A. (2024). When Eco-Anger (but Not Eco-Anxiety nor Eco-Sadness) Makes You Change! A Temporal Network Approach to the Emotional Experience of Climate Change. J. Anxiety Disord..

[38] Schwartz D., Loewenstein G. (2017). The Chill of the Moment: Emotions and Proenvironmental Behavior. J. Public Policy Mark..

[39] Innocenti M., Santarelli G., Lombardi G.S., Ciabini L., Zjalic D., Di Russo M., Cadeddu C. (2023). How Can Climate Change Anxiety Induce Both Pro-Environmental Behaviours and Eco-Paralysis? The Mediating Role of General Self-Efficacy. Int. J. Environ. Res. Public Health.

[40] Stanley S.K., Hogg T.L., Leviston Z., Walker I. (2021). From Anger to Action: Differential Impacts of Eco-Anxiety, Eco-Depression, and Eco-Anger on Climate Action and Wellbeing. J. Clim. Change Health.

[41] Pearce H., Hudders L., Van De Sompel D., Cauberghe V. (2021). Motivating Children to Become Green Kids: The Role of Victim Framing, Moral Emotions, and Responsibility on Children’s Pro-Environmental Behavioral Intent. Environ. Commun..

[42] Witte K., Allen M. (2000). A Meta-Analysis of Fear Appeals: Implications for Effective Public Health Campaigns. Health Educ. Behav..

[43] Ojala M. (2012). How Do Children Cope with Global Climate Change? Coping Strategies, Engagement, and Well-Being. J. Environ. Psychol..

[44] Ojala M., Lakew Y. (2017). Young People and Climate Change Communication. Oxford Research Encyclopedia of Climate Science.

[45] Schneider-Mayerson M., Leong K.L. (2020). Eco-Reproductive Concerns in the Age of Climate Change. Clim. Change.

[46] Rackin H.M., Gemmill A., Hartnett C.S. (2023). Environmental Attitudes and Fertility Desires among US Adolescents from 2005–2019. J. Marriage Fam..

[47] Hickman C. (2024). Eco-Anxiety in Children and Young People—A Rational Response, Irreconcilable Despair, or Both?. Psychoanal. Study Child.

[48] Owczarek M., Redican E., Shevlin M., Nolan E. (2025). Psychometric Assessment of Climate-Related Emotional Responses: A Systematic Review of Measures for Eco-Anxiety and Related Constructs. Curr. Psychol..

[49] Geraci A., Giordano G., Cucinella N., Cannavò M., Cavarretta M.V., Alesi M., Caci B., D’Amico A., Gentile A., Iannello N.M. (2024). Psychological Dimensions Associated with Youth Engagement in Climate Change Issues: A Person-Centered Approach. Curr. Psychol..

