# Supplementary material for: Hope and Fear: A Survey of Eco-Emotions and Climate Anxiety, Activism, and Well-Being Among Older Adolescents in Northern California
Source: Int J Environ Res Public Health. 2026 Jun 25;23(7):834. doi: 10.3390/ijerph23070834 (PMC13410137; doi:10.3390/ijerph23070834)
Supplement: Supplementary file 1 [file ijerph-23-00834-s001.zip › ijerph-4330192-supplementary.pdf]

Supplementary File S1

Table. Background characteristics and emotions when thinking about climate change (N=521)

	Overall sample, *n*(%)	Positive eco-emotions, *M (SD)*	Negative eco-emotions, *M (SD)*
Total	521	2.62 (0.55)	3.15 (0.63)
Age group			
17-19	419 (80.4)	2.64 (0.56)	3.13 (0.63)
20-24	102 (19.6)	2.55 (0.54)	3.22 (0.56)
Gender			
Female	408 (78.3)	2.61 (0.53)	**3.21 (0.59)**
Male	82 (15.7)	2.70 (0.64)	**2.80 (0.69)**
Non-binary	26 (5.0)	2.44 (0.62)	**3.38 (0.49)**
Race/ethnicity			
Asian American, Pacific Islander, Native American	168 (32.2)	2.61 (0.58)	**2.98 (0.61)**
White	116 (22.3)	2.59 (0.52)	**3.41 (0.56)**
Latinx	69 (13.2)	2.71 (0.55)	**3.13 (0.57)**

Black, African American	42 (8.1)	2.66 (0.64)	**3.06 (0.80)**
Multiracial, Other	122 (23.4)	2.58 (0.52)	**3.18 (0.57)**
Feelings about living on present income			
Very difficult	50 (9.6)	2.55 (0.60)	3.16 (0.60)
Difficult	110 (21.1)	2.59 (0.53)	3.13 (0.66)
Getting by	197 (37.8)	2.64 (0.56)	3.09 (0.66)
Living comfortably	159 (30.5)	2.63 (0.54)	3.24 (0.54)

Eco-emotions were assessed with a 6-item scale ranging from 1(lowest) to 4 (highest). Positive emotions included hopeful, faithful, empowered, motivated, supported, and determined ($\alpha = 0.80$). Negative emotions include angry, guilty, disgusted, frustrated, afraid, and nervous. Bold indicates statistically significant differences in eco-emotions at $p<.05$ within the demographic category ($\alpha = 0.85$).

Supplementary File S2

Exploratory Factor Analysis

A factor analysis of emotions reported when thinking about climate change was conducted using the Principal Components Analysis method of extraction. We first verified that the data were feasible for exploratory factor analysis using goodness-of-fit tests. Bartlett's test of sphericity was significant ($X^2[105] = 2648.047$, $p < .001$), indicating that the dataset was appropriate for factor analysis because all correlations within the correlation matrix were significant. Kaiser-Meyer-Olkin measure of sampling adequacy also indicated strong relationships among variables (KMO = 0.87). Varimax rotation was chosen to simplify the interpretation of factors with the goal of achieving clearly defined factor structures. The final results of the exploratory factor analysis are displayed in the table below.

Table. Results of Exploratory Factor Analysis

	**Factor**		
**Items**	1	2	3
Angry	**.841**	.061	-.105
Frustrated	**.806**	.080	-.191
Afraid	**.789**	.012	-.064
Nervous	**.783**	.023	-.129
Disgusted	**.710**	-.122	.010
Guilty	**.543**	.196	.156
Hopeful	-.106	**.789**	.048
Faithful	-.063	**.720**	.242
Empowered	.100	**.702**	.015
Motivated	.273	**.645**	-.363
Supported	-.033	**.642**	.123
Determined	.352	**.641**	-.366
Indifferent	-.249	.204	**.714**
Doubtful	.419	.047	**.551**
Interested	.466	.429	-.452

Notes: Extraction Method: Principal Components. Rotation Method: Varimax with Kaiser Normalization. Rotation converged in 5 iterations. Loadings larger than .50 are bold.

In the exploratory factor analysis, the first factor had an initial eigenvalue of 4.578 and explained 30.52% of the total variance in the data. The items in this factor featured negative emotions about climate change. The second factor had an initial eigenvalue of 2.83 and accounted for 18.92% of the variance. The items in this factor reflected positive emotions about climate change.

The third factor accounted for 8.48% of variance with an initial eigenvalue of 1.27. This factor included two items, but they were poorly correlated ($r = .09$, $p < .05$). The item "interested" had medium loadings on all three factors. These three items were not included in the eco-emotion measures used in this study.

Supplementary File S3

Survey Questions

Eco-Emotions [15,19,21]

- When thinking about climate change, how often do you feel the following emotions?
    - (1 = very, 2 = moderately, 3 = not very, 4 = not at all)

1. Interested
2. Angry
3. Hopeful
4. Disgusted
5. Doubtful
6. Indifferent
7. Frustrated
8. Empowered
9. Motivated
10. Determined
11. Faithful
12. Guilty
13. Supported
14. Nervous
15. Afraid

Eco-anxiety [15]

- Does climate change make you think any of the following?
    - (1 = yes, 2 = no, 3 = prefer not to say)

1. I won't have access to the same opportunities that my parents had
2. I'm hesitant to have children.
3. Humanity is doomed
4. The future is frightening
5. My family's security will be threatened (e.g. economic, social, physical security)
6. People have failed to take care of the planet
7. The things I value most will be destroyed
8. My concerns about climate change undermine my ability to work to my potential

Eco-impairment [15,16]

- Over the last 2 weeks, how often has thinking about climate-related events and other global environmental conditions affected the following activities?
  - (1 = not at all, 2 = some of the days, 3 = over half of the days, 4 = nearly every day)

1. Sleeping
2. Enjoying time with family and friends
3. Working or studying

Confidence in climate action [28,29]

- How much do you agree or disagree with the following statements?
  - 1 = strongly disagree, 2 = disagree, 3 = agree, 4 = strongly agree)

1. My actions have an influence on climate change.
2. My awareness of climate change or environmental damage has given me more appreciation for and a deeper connection to people and the planet.
3. I avoid finding out more about climate change or environmental damage.
4. Connecting with like-minded people around climate change has helped me feel empowered.
5. Active engagement in addressing climate change or environmental damage helps me cope with my distress.
6. I believe collective action will help combat climate change or environmental damage.
7. My actions to reduce the effects of climate change will encourage others to reduce the effects of climate change.
8. I wish I behaved more sustainably.

Climate Actions

- In the past 12 months, how often did you engage in the following activities?
  - (1 = never, 2 = rarely, 3 = sometimes, 4 = often)

1. Posted about the environment on social media
2. Made a choice to not buy a certain product
3. Talked to other people about environmental issues
4. Volunteered for environmental project (e.g. trail clean-up, planting trees/flowers)
5. Took a class related to climate change, environmental health, or a similar topic
6. Joined an environmental organization at the university
7. Joined an environmental organization outside of the university

8. Attended a town hall meeting and/or other local governmental meetings
9. Participated in advocacy related to the environment (e.g. joined a protest or demonstration, contacted politicians)
10. Kept up-to-date on the news about the environment
11. Read a book and/or watched something about the environment
12. Joined an environmental conference or summit (in-person or online)

<u>Children's Hope Scale [30]</u>

- These questions about your general well-being. Consider how often you identify with the following statements.
    - (1 = all of the time, 2 = a lot of the time, 3 = some of the time, 4 = a little of the time, 5 = none of the time)

1. I think I'm doing pretty well.
2. I can think of many ways to get the things in life that are most important to me.
3. I am doing just as well as other people my age.
4. When I have a problem, I can come up with lots of ways to solve it.
5. I believe the things I have done in the past will help me in the future.
6. Even when others want to quit, I know that I can find ways to solve the problem.
7. I am hopeful about the future.

<u>Compassion [31]</u>

- How much do you agree or disagree with the following statements?
    - (1 = strongly disagree, 2 = disagree, 3 = agree, 4 = strongly agree)

1. It's important to take care of people who are vulnerable.
2. When I see someone hurt or in need, I feel a powerful urge to take care of them.
3. I often notice people who need help.
4. I am a very compassionate person.
